# Measured GFR in murine animal models: review on methods, techniques, and procedures

**DOI:** 10.1007/s00424-023-02841-9

**Published:** 2023-08-08

**Authors:** Silvia Teixido-Trujillo, Sergio Luis-Lima, Marina López-Martínez, Maruja Navarro-Díaz, Laura Díaz-Martín, Elia Escasany-Martínez, Flavio Gaspari, Ana Elena Rodríguez-Rodríguez

**Affiliations:** 1 Universidad de La Laguna, Faculty of Medicine, San Cristóbal de La Laguna, Spain; 2 Research Unit, Hospital Universitario de Canarias, San Cristóbal de La Laguna, Spain; 3 Department of Laboratory Medicine, Complejo Hospitalario Universitario de Canarias, San Cristóbal de La Laguna, Spain; 4 Department of Nephology, Hospital Universitari Vall d’Hebron, Barcelona, Spain; 5 Department of Nephology, Hospital de Sant Joan Despí Moisès Broggi, Barcelona, Spain; 6 Instituto de Tecnologías Biomédicas (ITB), Universidad de la Laguna, San Cristóbal de La Laguna, Spain; 7 Lipobeta group. Departamento de Ciencias Básicas de la Salud, Facultad de Ciencias de la Salud, Universidad Rey Juan Carlos, Madrid, Spain; 8 Instituto di Ricerche Farmacologiche Mario Negri (IRCCS), Clinical Research Center for Rare Diseases ‘Aldo & Cele Daccò, Bergamo, Italy

**Keywords:** Glomerular filtration rate (GFR), Renal function, Kidney damage, Animal models, Exogenous markers

## Abstract

Chronic kidney disease (CKD) is one of the most common chronic diseases worldwide, with increasing rates of morbidity and mortality. Thus, early detection is essential to prevent severe adverse events and the progression of kidney disease to an end stage. Glomerular filtration rate (GFR) is the most appropriate index to evaluate renal function in both clinical practice and basic medical research. Several animal models have been developed to understand renal disease induction and progression. Specifically, murine models are useful to study the pathogenesis of renal damage, so a reliable determination of GFR is essential to evaluate the progression of CKD. However, as in clinical practise, the estimation of GFR in murine by levels of serum/urine creatinine or cystatin-C could not be accurate and needed other more reliable methods. As an alternative, the measurement of GFR by the clearance of exogenous markers like inulin, sinistrin, ^51^Cr-EDTA, ^99m^Tc-DTPA, ^125^I-iothalamate, or iohexol could be performed. Nevertheless, both approaches—estimation or measurement of GFR—have their limitations and a standard method for the GFR determination has not been defined. Altogether, in this review, we aim to give an overview of the current methods for GFR assessment in murine models, describing each methodology and focusing on their advantages and limitations.

## Introduction

Chronic kidney disease (CKD) is a progressive disease characterized by a gradual loss of kidney function over time [17, 42, 59]. CKD can be asymptomatic until late stages; so early detection is essential to prevent disease progression and related adverse events [22, 25]. Several aspects of the pathogenesis of CKD are not completely elucidated. In this regard, murine models are useful to study the pathogenesis of renal damage and in particular to understand the molecular background of disease progression in CKD [13, 31, 63].

Glomerular filtration rate (GFR) is considered the best index of renal function [15, 23, 53]. Accordingly, a reliable evaluation of GFR is important both in the clinics and in animal models of disease. In murine models, the assessment of GFR is useful to evaluate the degree of renal dysfunction, disease progression, and benefits of drugs designed to treat renal damage. In mice and rats, renal function can be estimated by the levels of serum creatinine and by 24 h creatinine [64]. On the other hand, GFR can be directly measured by the clearance of exogenous markers like inulin, sinistrin, ^51^Cr-EDTA, ^99m^Tc-DTPA, ^125^I-iothalamate, or iohexol [1, 4, 27, 40]. Both approaches—estimation and measurement of GFR—have advantages and disadvantages. The estimation is simple but not always reliable and the measurement is reliable but cumbersome. In this review, we will describe the available methods and techniques used to measure kidney function in murine models with a special focus on their applicability, utility, advantages, and disadvantages.

## Characteristics of a marker of renal function

To be used as a marker of GFR, a molecule must fulfill several criteria, such as: to have a constant production, not to be bound to proteins or degraded by metabolism, be freely filtered by the glomerulus, and have no interaction with renal tubular cells i.e. secretion or re-absorption. The measurement of the clearance of such a compound allows a reliable GFR evaluation. However, no endogenous substance meets all these characteristics simultaneously, as will be discussed later in the review. Conversely, a number of exogenous substances, such as fructose polymers (inulin, sinistrin), metal chelates (^51^Cr-EDTA, ^99m^Tc-DTPA), and tri-iodobenzene derivatives (e.g. iohexol, iothalamte, iopamidol, iopromide), have been proven to act as an ideal marker.

## Estimation of GFR based on creatinine

### Serum creatinine

It is the most used endogenous marker used to estimate renal function in clinical practice and research, as well as in animal models [11, 37]. Creatinine is a waste product of normal muscle metabolism, an organic compound derived from the breakdown of creatine, a nitrogenous organic acid synthesized in the liver and located mainly in the muscles. Creatinine is not bound to proteins and is freely filtered by glomeruli [61] but its synthesis is not constant since it is determined by the daily intake of protein and muscle turnover. Moreover, there is relevant renal tubular handling of the molecule i.e. secretion and reabsorption [7, 12]. All these conditions limit the utility of creatinine as a marker of renal function. In particular, in mice, creatinine tubular secretion may reach 50% of total creatinine clearance, reducing plasma creatinine levels and consequently leading to the overestimation of GFR [12]. Also, gender differences in tubular secretion have been described: males may secrete more creatinine than females [12]. This may lead to flaws in the estimation of GFR between male and female animals [12]. Vallon et al. have demonstrated that OAT3 (organic anions transporter isoform 3) contributes to this tubular creatinine secretion in rodents [58]. In addition, kidney disease promotes creatinine tubular secretion, which may mask the reduction in GFR during the evolution of renal disease [7, 12]. These considerations must be taken into account when using creatinine as a marker of renal function in mice models.

**Fig. 1:**
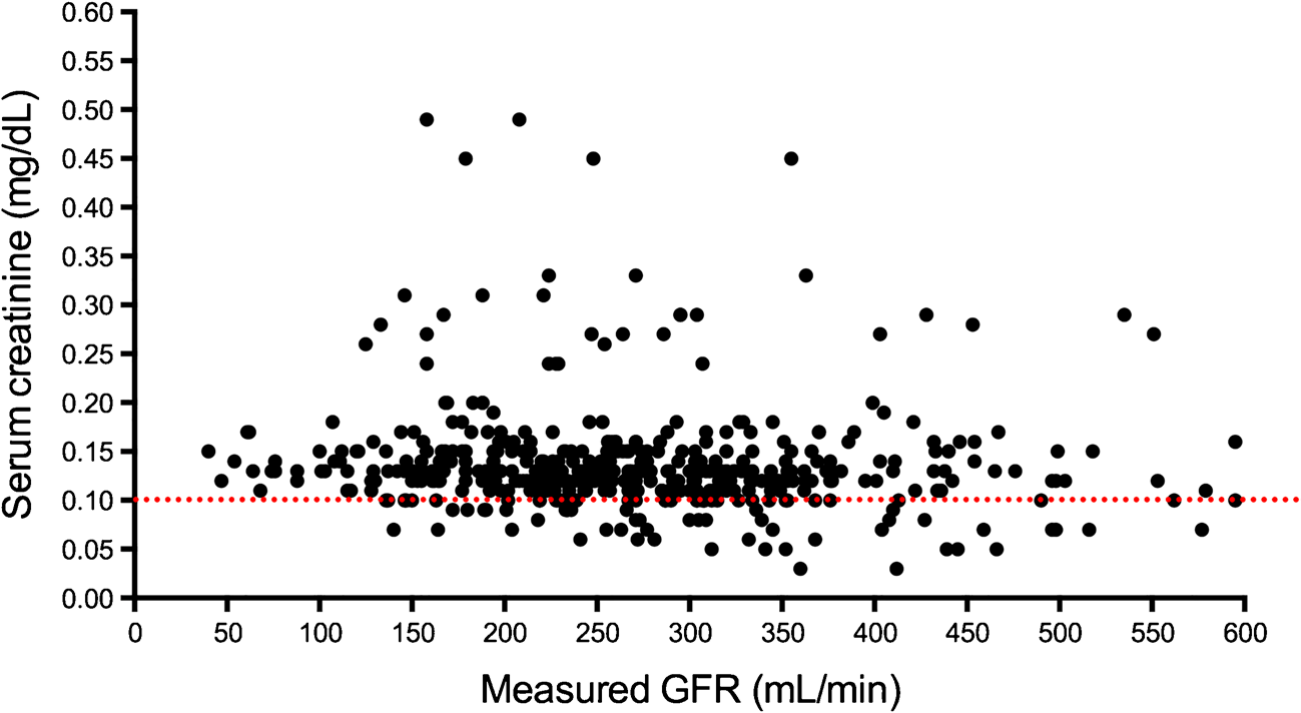
Relationship between serum creatinine and measured glomerular filtration rate in C57BL/6J mice (*n*=180). Measured glomerular filtration rate (GFR) was assessed by plasma clearance of iohexol – Dried blood spot (DBS). Serum creatinine was measured by an automated enzymatic method in the Cobas 8000 equipment (Roche Diagnostic)

Also, the measurement of creatinine has largely been challenging. The first method, developed in 1886, to measure creatinine was the alkaline picric Jaffé reaction (colorimetric method) [18]. The interference of this method with chromogens i.e. bilirubin, glucose, or hemoglobin has led to inaccuracies in humans [50, 56]. In rodents, non-specific chromogens may cause a five-fold overestimation of creatinine [11, 28]. Several alternative methods have been adapted to measure serum creatinine. Dunn et al. developed an HPLC (high-performance liquid chromatography) assay able to measure low levels of creatinine in 25uL of plasma in mice and correlated the creatinine clearances with inulin-based clearances [11]. Later, Yuen et al. simplified this method using a smaller sample volume without acid addition in the acetonitrile precipitation step [64]. Thus, HPLC has been recommended as a precise method to measure creatinine in mice. However, the HPLC assay is time-consuming and expensive and so, has been rarely used. The enzymatic determination, which is considered nowadays as the reference method in rodents, was validated in 2007 [19]. This method is based on a cascade of reactions with the aid of creatininase, creatinase, and sarcosine oxidase and has good agreement with the method using HPLC [19]. However, despite these improvements in the measurement techniques, the intrinsic limitations of creatinine, i.e. tubular secretion, dependence on muscle metabolism, still remain. For the scope of this review, we measured serum creatinine in 180 mice that underwent measured GFR by the plasma clearance of iohexol-DBS technique [40] (Fig. 1). The agreement between creatinine and measured GFR was weak. In fact, a single value of serum creatinine was associated with a wide range of GFR. For example, a value of 0.10 mg/dL of creatinine is associated with a wide range of GFR from 150 to 600 μl/min, indicating about 400% variability (Fig. 1). Of note, this error is larger than in humans, where the variability between creatinine and measured GFR was reported to be as large as 200% [37]. All the above indicates that creatinine itself is far from being an ideal marker of GFR leading to relevant over and underestimation of real renal function in rodents. Clearly, this may jeopardize research in animal models of renal disease.

### 24-h creatinine clearance

This method has been widely used to assess GFR in animal models. However, it must be remembered that the limitations of serum creatinine as a marker of renal function (Fig. 1*)* affect the precision and accuracy of the 24-h collection in reflecting renal function [7, 12, 58]. Urine sample collection is time-consuming and stressful for the animals, both facts that may affect the evaluation of renal function. Rodents must be kept for 2-3 days in metabolic cages before starting the 24-h collection [54] to acclimate. Finally, blood samples are needed to measure serum creatinine. For blood extraction animals may be sedated or anaesthetized depending on different protocols [7, 8] to minimize animal stress. The clearance is calculated as the urine concentration of creatinine multiplied by the volume of urine during 24-h and divided by the concentration of creatinine in serum.

All the above indicates that creatinine is not an ideal marker of GFR in rodents. The agreement between creatinine and real renal function is very poor and the variability is high. Thus, in terms of investigation, creatinine and creatinine clearance are not reliable methods to evaluate GFR in rodents. The limitations of creatinine pertain more specifically to models aimed at studying CKD than to those of AKI. CKD is characterized by a progressive and gradual loss of kidney function over time. During this period, the kidneys can undergo various adaptive responses to maintain overall function like hypertrophy and hyperfiltration, which can partially mask the rise in serum creatinine levels. This could result in delayed or less pronounced increases in creatinine, making it a less sensitive marker for detecting early stages of CKD. On the other hand, AKI involves a sudden and severe loss of kidney function. The decline in renal function occurs rapidly, reducing the time of compensatory mechanism to mask the rise in creatinine levels. Thus, creatinine is generally considered a more sensitive marker for detecting AKI compared to CKD.

## Estimated GFR by cystatin-C

Cytatin-C (CysC) is a low molecular weight (13KDa) protein of the super-family of cysteine protease inhibitors. It is produced at a constant rate by a housekeeping gene that is present in all nucleated cells. Cystatin-C is freely filtered across the glomerulus. However, it is reabsorbed and metabolized by tubular epithelial cells, with precludes the use of 24-h urine collection for clearance analysis. Also, CysC levels are affected by age, male, height, subclinical inflammation, central adiposity, diabetes, and metabolic syndrome, among others [20, 39, 55]. Even so, in animal models, CysC has been used to determine renal damage. Song S et al. were the first to use cystatin-C as a reliable method to measure renal function in mice models. In this study, they compared the sensitivity of creatinine, urea nitrogen (BUN), and CysC in the detection of acute renal failure in mice. They observed that CysC was more sensitive than the others [52]. Worner S et al. used plasma CysC levels to determine aprotinin-associated kidney damage [60]. Leelahavanichkul A et al. determined that CysC was a better early detection biomarker of renal damage than creatinine in sepsis. However, this result was influenced by non-renal factors limiting the accurate prediction of GFR in sepsis animal models [21]. Thus, CysC would not be an ideal marker to measure renal damage since there are non-renal factors that cause its modification. Even so, CysC has advantages since it is easy to measure and very sensitive to ELISA measurement [62].

## Measuring GFR by an exogenous marker

### Clearance of inulin

Inulin is a polymer of fructose with a molecular weight of 5200 Da [15]. It is found in plants like chicory and garlic, among others that use inulin as energy. Since the pioneering studies of Homer Smith in 1951 the urinary and plasma clearances of inulin have been considered a classic method to measure renal function in humans [51]. Later, inulin has also been considered as the reference method to measure GFR in rodents. In humans and animals, inulin is not metabolized, is bound to plasma proteins, and is freely filtered by the glomeruli without being reabsorbed or secreted by tubular cells [49].

Despite these major advantages, the method has two major limitations: the cost of inulin is high and the pre-analytical procedures to measure the molecule are cumbersome. Inulin can be used un-labelled or labelled with radioactive markers (^3^H or ^14^C) or fluorescein-isothiocyanate (FITC). In any case, the solubility in water of inulin is poor and requires extensive treatment to prepare the solution for injection. The molecule must be dissolved in saline, filtered, heated at high temperatures, and dialyzed overnight to remove low-weight fragments inulin and residual free radioactive markers. Finally, the solution is injected either in a single intravenous bolus [41] or continuous infusion [6, 26] and plasma and/or urine are collected at different time points to calculate the clearance. Animals must be anaesthetized; bladder catheterization is needed to collect urine and several blood samples are required. Then, un-labelled inulin is measured by HPLC whereas, for radioactive-labelled inulin, a count emitting isotope radioactive is needed. All these steps make the technique not only cumbersome but also prone to errors, a fact that has not frequently been considered before.

**Fig. 2:**
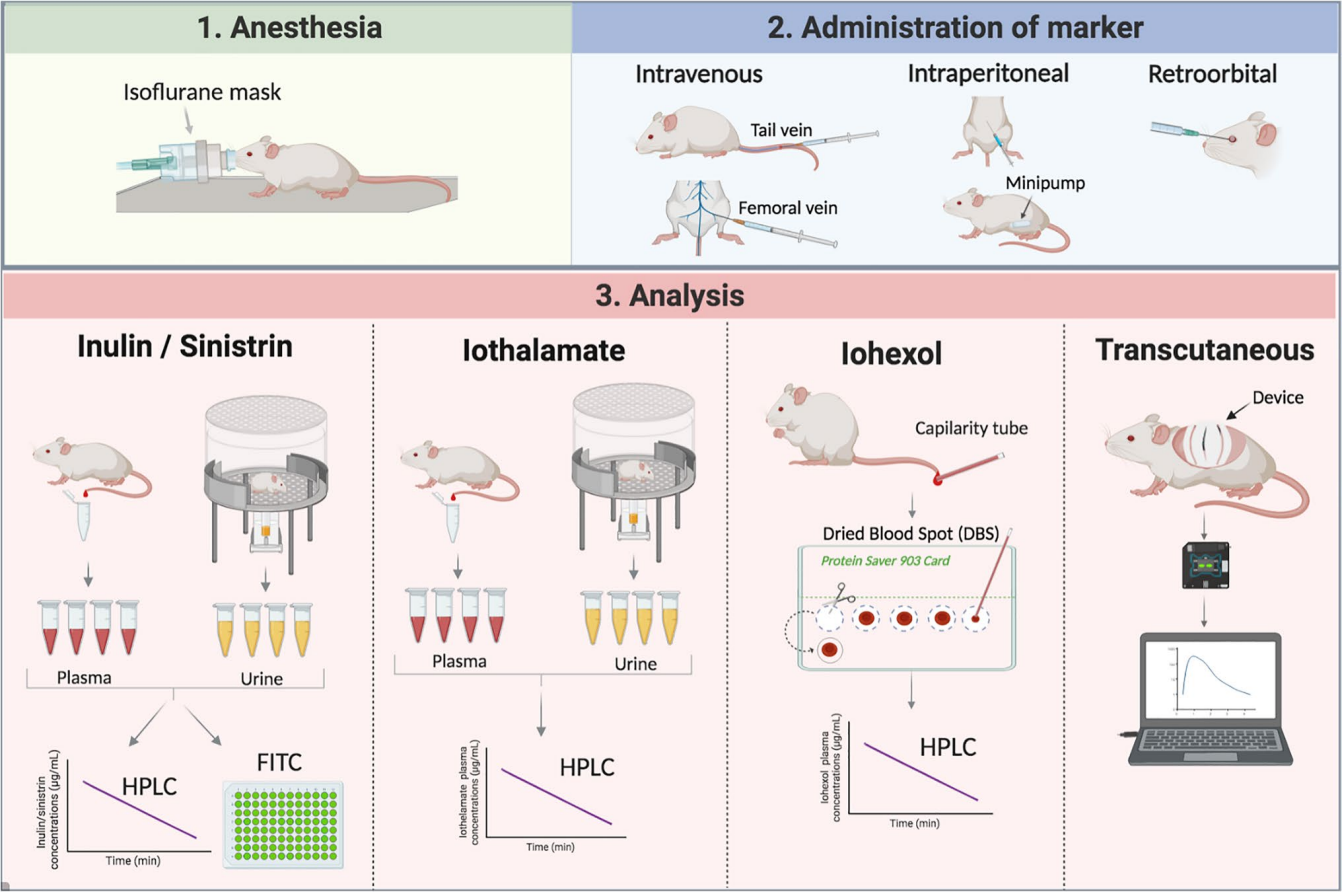
Schematic representation of the main methods (exogenous markers) to measure renal function in rodents. All of them have three steps: (1) The animal must be anaesthetized or sedated for (2) the administration of the tracer or the placement of the measurement device in the animal, and the last step (3) is the analysis that will be different depending of the marker used

In 2003, Qi et al. published the use of FITC-inulin as a method to measure GFR in mice [38]. Dialyzed FITC-inulin was injected retro-orbitally under light anaesthesia, and then, 20 μL of blood was collected via the saphenous vein at a different time point in conscious animals. Then, a fluorometer was used to determine fluorescence in blood and urine. Clearly, this approach is simpler than standard inulin. Nevertheless, FITC-labelled inulin has the same requirements for the preparation solution for injection i.e. the molecule has to be dissolved, filtered, heated at high temperature, dialyzed overnight, and filtered again. Thus, independently of the method used, i.e. inulin alone, bound to radioactive isotopes or fluorescein, the whole procedure is tedious and laborious *(*Table 1, Fig. 2)*.*

**Table 1:** Comparison of different methods to measure GFR in rodents

Methods	Rodent preparation	Markers preparation	Injection	Blood samples	Urine samples	Instrument	Measurement	Limitations
Inulin or FITC-Inulin	Anaesthesia during all the procedure	Filtered, heated high temperatures, dialyzed. FITC-inulin	i.v.; r.o.; i.p.	Yes	Yes	HPLC; Fluorometer;	rGFR or eGFR	High inulin cost, cumbersome pre-analytic process
Sinistrin or FITC-sinistrin	Anaesthesia only for the injection	Sinistrin FITC-sinistrin	i.v.; r.o.; i.p.	Yes	Yes, sinistrin No	Fluorometer	eGFR	Estimation and not direct measurement
Transcutaneous	Mice shaved 24 h before procedure. Anaesthesia	FITC-Sinistrin. Device: battery, adhesive patch.	i.v.; r.o.; i.p.	No	No	Fluorometer	eGFR, using a conversion factor	Not direct measurement, low precision, and accuracy, high cost
Radioactive	Anaesthesia	^51^Cr-EDTA; ^99m^Tc-DTPA	i.v.	Yes	Yes	Gamma counter	rGFR	Unsafely and difficult to manage
Iothalamate	Anaesthesia during all the procedure	-	i.v.	Yes	Yes	HPLC	rGFR	Continuous infusion of marker and sampling from a cannula
Iohexol (DBS)	Sedation only for the injection	In mice: dilution of iohexol in physiological serum	i.v.	Yes	No	HPLC	rGFR	Do not seem to have any limitation

*i.v.,* intravenous; *r.o.,* retro-orbital; *i.p*., intraperitoneal; *HPLC*, high-performance liquid chromatography; *FITC*, fluorescein-isothiocyanate. *rGFR*, real glomerular filtration rate; *eGFR*, estimated glomerular filtration rate

### Sinistrin: the new inulin?

Sinistrin is a polyfructosan extracted from the roots of *Urgea maritime* and has a lower molecular weight (3500 Da) than inulin. To overcome the poor water solubility of inulin, sinistrin was introduced. Sinistrin, like inulin, can be used and labelled as fluorescein and most studies using sinistrin are complex to FITC. Sinistrin and FITC-sinistrin are highly soluble in aqueous solvents at room temperature and do not require heating to prepare the solution injection (Table 1; Fig. 2). Furthermore, it does not need to be dialyzed to remove non-dissolved fragments. Thus, FITC-sinistrin has been proposed as a simpler method to measure GFR [34–36]. Also, FITC-sinistrin has been even adapted to be measured by transcutaneous devices [44].

### Transcutaneous methods for GFR measurement

In these methods, a molecule with a fluorescent marker is injected and the determination of GFR relies on the lecture of the change in the fluorescence over time by a device placed over the skin of the animal. Most of the available studies use FITC sinitrin as the exogenous marker. The decay of fluorescence is used to calculate the half-life of the elimination of the marker and then, you need conversion half-life data into a GFR (mL/min) because the transcutaneous method results in relative emission signals which do not allow conversion into concentration using standard curves. The main advantage of these methods is that they are non-invasive (Table 1, Fig. 2). Schock-Kusch et al. reported its use for the first time in rats in 2009 [44]. In this study, a femoral vein and artery were catheterized in animals for the injection of FITC-sinistrin and blood sampling, respectively. Animals were anaesthetized and the fluorescence was tested with a small imager placed over a depilated ear. Simultaneously the plasma clearance FITC-sinistrin was measured using an enzymatic method and this clearance was used to calculate the conversion factor as follows: GFR (mL/min/100g b.w) = 31.26 (mL/100g b.w.) / t_1/2_ (FITC-S) (min) determined by the transcutaneous method. In 2011, the same group optimized the technique using an optical device fixed on a depilated region on the back of the rat. This device was built up from a light-emitting diode with an emission maximum for FITC at 480 nm, a photodiode that detects the emitted light at 520 nm. Also, the device has an internal memory to save the information. Before injection, the device detects a background signal and after injection, the sampling rate is about 60 measurements per min. In 2012, the same group implemented this technique in conscious mice and recalculated the conversion factor as 14616.8 (μL/100g b.w.) [46].

This technique has been used in diverse mice models such as healthy mice, nephrectomised mice, and animals with nephronophthisis [46]; C57BL/6, Balb/c, SV129, NMRI mice [43] lean and obese C57BL/6J mice [29] and animals lacking renal angiotensin-converting enzyme (ACE) [16]. Also, it was used in rat models like healthy rats as well in those with unilateral nephrectomy, 5/6 nephrectomy or rats with cystic kidney diseases [44] or the Dahl salt-sensitive rats [5].

However, the major limitation of this technique is that it is an indirect method for measuring GFR. The marker excretion kinetics is evaluated by the analysis of the change in the relative fluorescence over time, and not as the decrease in the absolute concentrations of the marker. Thus, conversion factors are needed to estimate GFR values, which may lead to uncertainty in GFR results. Maybe, due to that limitation, diverse articles showed low precision and accuracy between GFR measured with transcutaneous devices versus the methods using plasma samples. In rats, Schock-Kusch et al. found a weak correlation, i.e. *r*^2^= 0.59 and low limits of agreement from 0.3 to −0.3 ml/min/100g [45]. In mice, a poor agreement for the two-compartment analysis was also reported: *r*^2^ = 0.33 with wide limits of agreement from 582 to −609.1 μL/min/100g [46]. A similar bias was found comparing five groups of male mice [43]. Moreover, a recent study compared GFR measurements by transcutaneous and plasma methods in obese and lean animal mice without showing a clear correlation between the methods in obese animals [5]. Thus, this approach may not be useful for all types of diseases. Another limitation to consider is the high cost of the device (1000$) and the low durability of its battery (only 2 h), which makes it unfeasible for studies with a high number of animals. The major advantage of this method is its independence of blood/urine sampling and laboratory assays allowing the evaluation of renal function almost in “real-time”. Future device optimization could make this technique an effective method to GFR measure in conditions where high precision and accuracy are not required.

### Radiolabelled tracers

Since the 1970s, the use of radioactive markers has been used to measure GFR both in clinical practice animal models. Although frequently used in humans, few groups have worked with these methods in animal models. The two most used radiolabelled markers are ethylenediaminetetraacetic acid with radioactive chromium-51 (^51^Cr-EDTA) and diethylene triamine pentaacetic acid with radioactive technetium-99 (^99m^Tc-DTPA). ^51^Cr-EDTA has a molecular mass of 292 Da, and ^99m^Tc-DTPA has a molecular mass of 393 Da and, due to their low molecular mass, are freely filtrated by glomerulus [48]. The method consists of measuring plasma and urine clearance of single injections of these radiolabelled compounds through the tail vein or using intraperitoneal injections [30]. Then, blood and urine samples are taken at several time points and the marker is counted using a gamma counter and GFR is calculated.

^99m^Tc-DTPA has been used in healthy male Wistar rats as well as in animals with chronic kidney disease or nephritic syndrome induced by doxorubicin [30]. The major limitation of this technique is derived from the use of radioisotopes. Radiolabelled reagents require special licensing and are difficult to store; the waste is difficult to handle and dangerous for staff who are exposed to it. Another limitation is that ^99m^Tc can dissociate from DTPA and that up to 13% of ^99m^Tc-DTPA can bind to plasma proteins, which translated into an underestimation of GFR [9]. Another limitation of ^9m^Tc-DTPA is that results seem to vary greatly depending on the distributor of the radiolabelled isotope. Moreover, the main manufacturer of ^51^Cr-EDTA in Europe has recently ceased its distribution [1]. Therefore, when possible other methods should be preferred to measure GFR in rodents.

### Non-radiolabelled contrast media

#### Iothalamate

Iothalamate is an ionic contrast, derived from the tri-iodobenzoic acid with a molecular weight of 637 KDa. This molecule has been seldom used for GFR assessment in murine models. Bell et al. developed a rapid and sensitive HPLC method to detect iothalamate and para-aminohippuric acid in the serum and urine of rats [2]. The analytical assay allowed a reliable and simultaneous measurement of both GFR and renal blood flow. However, the procedure suffered from two important drawbacks. Firstly, a surgical preparation of the animals was required followed by a recovery period from anaesthesia before starting the intravenous administration of the markers by continuous infusions in the jugular vein. Secondly, sampling was performed from a cannula in the carotid artery and urine collection. For these reasons, the procedure could be considered difficult and cumbersome.

#### Iohexol/iohexol-DBS

Iohexol (Omnipaque™, GE Healthcare) is a non-radioactive, iodinated, non-ionic monomeric, and water-soluble molecule widely used as a contrast medium [24, 32]. In comparison with the first generation of contrast agents, iohexol has lower osmolarity and toxicity and better safety. Iohexol is excreted un-metabolized by glomerular filtration, without being reabsorbed or secreted by renal tubular cells [27]. Neither hepatic metabolism nor interaction with blood cells has been described [3, 10]. In humans, the use of the clearance of iohexol as a reference method to measure GFR has been established almost 30 years ago [3, 14].

During the last decade, some groups implemented the clearance of iohexol in rodents. In general, the method consists of the i.v. injection of a single dose of iohexol, followed by the extraction of several blood samples from the tip of the tail to perform the pharmacokinetic analysis. Iohexol is measured by chromatographic analysis using HPLC. *Schulz et al.* described the plasma clearance of iohexol in rats in 2014 [47] using liquid-chromatography-electrospray-mass-spectrometry (LC-ESI-MS). They used male HsdRCCHan:WIS rats, which were administered with different doses of iohexol by tail vein, and the animals were sacrificed at different time points after iohexol infection (15, 30, 60, and 90 min) to obtain blood samples. Passos et al. validated the plasma clearance of iohexol in rats [33] versus the “classic” gold standard, the clearance of inulin, using capillary electrophoresis. They observed a correlation between iohexol clearance and inulin clearance (*r*=0.792). However, the procedure required a large volume of blood because the molecule was determined in plasma or serum instead of blood and the animals were handled under anaesthesia. Later, Carrara *et al*. developed a feasible, safe, and reliable protocol to determine the GFR in conscious rats [4]. A single intravenous injection of iohexol (129.4 mg) was administered and only 4 blood samples of just 15 μL were drawn from the tail vein at different times from the injection (20, 40, 120, and 140 min. In line with this study, Luis-Lima et al. described a simplified method in conscious mice [27]. In brief, 6.47 mg of iohexol was injected intravenously into the tail vein of sedated mice. Then, reduced volumes of blood samples (10μL per point) were collected at 15, 35, 55, and 75 min after injection from the tip of the tail in conscious and unrestricted mice. Finally, iohexol was measured by HPLC-UV in total blood applying a corrector factor of 0.89. The results of the reference (two-compartment model) and simplified method (one-compartment model) were comparable. This approach was tested in mice with different levels of renal function, from normal GFR to animals with a heminephrectomy and a model of CKD [27]. This approach uses a limited volume per sample (10μL), a relevant aspect for the mice that also allows the repetition of the test to evaluate renal function changes over time. In 2021, Rodríguez-Rodríguez AE et al. further simplified the procedure by the use of dried blood spot (DBS) sampling without losing accuracy and precision [40]. Blood samples from the tip of the tail were collected using heparinized capillary tubes of 5μl at different time points: 15, 30, 45, 60, and 75 min after injection, deposited on filter paper (Whatman 903, GE Healthcare, Cardiff, UK) and then allowed to dry for at least 24h. The total amount of blood taken is very low (25μL), which is a specific advantage of the DBS approach. Then, the extraction of iohexol from the filter paper was with 5% perchloric acid, ultrasonicated, and centrifuged [40]. The iohexol blood concentration from DBS samples was determined by HPLC (Table 1, Fig. 2). This DBS method was validated with the reference method in plasma, showing high accuracy and precision (concordance correlation coefficient (CCC) was 0.996). Taken together, the DBS technique represents a methodological simplification to determine GFR in small rodents improving animal welfare according to Russell and Burch’s 3Rs model for animal research without losing precision. *Turner* et al. validated a two-sample iohexol plasma clearance method in rodents to evaluate an early decline in GFR compared to inulin method and endogenous markers such as creatinine, urea, and cystatin-C [57]. After a single intravenous injection of 25 mg/kg of iohexol, blood samples were taken at 11 time points (2, 5, 10, 20, 30, 60, 90, 120,180, 240, and 300 min). Surprisingly, the GFR result obtained from the analysis of two samples (30 and 90 min) was closely approximated at the 11 samples clearance, with very high accuracy and almost no loss of precision. This way, the authors showed a simplified two-sample determination of iohexol as a feasible routine method to evaluate GFR. However, such a limited number of samples may lead to high errors in GFR estimation due to potential random errors in iohexol determination. Conversely, blood timing (which seems very crucial in this 2-point procedure) and/or blood drawing errors resulting in “outsiders” points may easily identified and ruled out in a multiple points procedure, thus leading to a more reliable GFR assessment.

## Conclusions

GFR measurement has been intensively studied in both humans and animals with a common goal: to seek out an affordable, reproducible, simple, rapid, safe, and comfortable GFR procedure. This task has been proven to be particularly demanding in small animals (rodents). The need to overcome the difficulties related to urine collection and reducing the amount of blood samples and the need to avoid the use of radiolabelled substances to improve the safety of both animals and laboratory personnel were the most relevant reasons that acted, along these last years, as driving forces toward the miniaturization of the procedure. As shown in Table 1, the methods to measure GFR in animal models are diverse and all of them have advantages or disadvantages. The endogenous markers, like creatinine or cystatin-C, are inaccurate and the exogenous markers have been developed to replace them. The radiolabelled markers (^99m^Tc-DTPA and ^51^Cr-EDTA) are cheap but not safe and should be replaced by another method. Inulin is considered the gold standard to measure GFR and is widely used but it is operationally complex and expensive. Iothalamate is less accurate than inulin but more affordable and easier to use. Iohexol is considered to be a more accurate and reliable method. It is a safe method and has been validated in different animal models of rats and mice. Fluorescence markers like FITC-inulin or FITC-sinistrin, are widely used because are considered to be safe and effective. Moreover, FITC-sinistrin is used as a marker in the transcutaneous method to measure GFR. This method is non-invasive, which may reduce the stress on the animal and eliminate the need for blood sampling. Additionally, this method has the potential to allow for real-time monitoring of GFR changes in animals. Overall, while the transcutaneous method of measuring GFR in rodents shows promise, further research is needed to establish its accuracy and reproducibility, and it should be used in conjunction with other established methods to ensure the most accurate and reliable measurement of GFR.

In conclusion, the choice of method for GFR measurement in rodents should depend on the specific research question, available resources, expertise of the researcher, and the safety and welfare of the animals. It is important to remember that any procedure performed on animals must be performed ethically and justified by scientific research.

## Data Availability

Not applicable
